# Impact of intra-abdominal pressure on early kidney transplant outcomes

**DOI:** 10.1038/s41598-022-06268-7

**Published:** 2022-02-10

**Authors:** Armando Coca, Carlos Arias-Cabrales, María José Pérez-Sáez, Verónica Fidalgo, Pablo González, Isabel Acosta-Ochoa, Arturo Lorenzo, María Jesús Rollán, Alicia Mendiluce, Marta Crespo, Julio Pascual, Juan Bustamante-Munguira

**Affiliations:** 1 Department of Nephrology, Hospital Clínico Universitario, Avda. Ramón y Cajal 3, 47003 Valladolid, Spain; 2 Department of Nephrology, Hospital del Mar, Paseo Marítimo de la Barceloneta 25-29, 08003 Barcelona, Spain; 3 Department of Nephrology, Hospital General, C/ Luis Erik Clavería Neurólogo s/n, 40002 Segovia, Spain; 4 Department of Cardiac Surgery, Hospital Clínico Universitario, Avda. Ramón y Cajal 3, 47003 Valladolid, Spain

**Keywords:** Biomarkers, Medical research, Risk factors

## Abstract

Increased intra-abdominal pressure (IAP) is common among post-surgical patients and may cause organ dysfunction. However, its impact after kidney transplantation on early postoperative complications and graft recovery remains unclear. We designed a prospective, observational cohort study to describe the prevalence and determinants of IAP, as well as its effect on delayed graft function, postoperative complications, and graft recovery. IAP was measured in 205 kidney transplant recipients every 8 h during the first 72 h after surgery using the urinary bladder technique. Intra-abdominal hypertension was defined as IAP ≥ 12 mmHg. Patients were followed for 6 months or until graft failure/death. Mean IAP was 12 ± 3.3 mmHg within the first 24 h. 78% of subjects presented with intra-abdominal hypertension during the first 72 h. Increased IAP was associated with higher renal resistive index [r = 0.213; *P* = 0.003] and lower urine output [r =  − 0.237; *P* < 0.001]. 72 h mean IAP was an independent risk factor for delayed graft function [OR: 1.31; 95% CI: 1.13–1.51], postoperative complications [OR: 1.17; 95% CI: 1.03–1.33], and absence of graft function recovery [HR for graft function recovery: 0.94; 95% CI: 0.88–0.99]. Increased IAP was highly prevalent after transplantation and was independently associated with delayed graft function, postoperative complications, and absence of graft function recovery. Routine IAP monitoring should be considered post-transplantation to facilitate early recognition of relevant complications.

## Introduction

In 2018, 21,227 kidney transplantations were performed in the European Union (3313 of them in Spain)^1^. However, the rising prevalence of chronic kidney disease produces a continuous increase in the allograft demand/supply ratio^2^. Early recognition of complications should be key to maximize the longevity of each organ, improving long-term results and reducing future demand for new allografts. In fact, postoperative complications are common after kidney transplant surgery; delayed graft function (DGF) can affect up to 30% of all deceased-donor transplant recipients^3^, renal artery or vein thrombosis produces 45% of early graft losses^4^, while bleeding that requires further intervention occurs in 4.4% of patients^5^.

Intra-abdominal pressure (IAP) is defined as the steady state pressure concealed within the abdominal cavity, according to the 2013 World Society of the Abdominal Compartment Syndrome guidelines (WSACS)^6^. Intra-abdominal hypertension (IAH) and abdominal compartment syndrome are clinical entities characterized by a sustained increase of IAP above normal values, which, in healthy subjects are < 5–7 mmHg. The abdominal compartment syndrome is a sustained elevation of IAP > 20 mmHg with new organ dysfunction^6,7^.

IAH and abdominal compartment syndrome are common among the critically ill^8–12^, associating higher rates of complications and mortality and longer hospital stay^13–15^. IAH has also been established as an independent cause of acute kidney injury following abdominal surgery in mixed intensive care unit (ICU) populations^16–18^. Depending on the context, incidence and prevalence rates of IAH/abdominal compartment syndrome vary, ranging from 14 to 83% in the case of IAH and between 0–56% for abdominal compartment syndrome^19^. Obesity, sepsis, abdominal surgery, or large volume fluid resuscitation have been correlated with higher risk of IAH/abdominal compartment syndrome^20–22^.

Moreover, the severity of IAH has been linearly correlated with renal artery resistive index (RRI) both in experimental models and humans^23,24^. In subjects with hepatorenal syndrome and tense ascites, paracentesis was associated with a significant decrease in IAP and RRI and increased renal blood flow, creatinine clearance and urine output^25^. In kidney transplantation, RRI is a strong predictor of DGF, especially when measured in the early postoperative period^26^.

Despite IAH is a widely recognized cause of kidney injury and death, its impact on kidney transplant outcomes has not been completely characterized. Recently, the impact of IAP changes on outcomes after kidney transplantation, specifically third-day serum creatinine (SCr), DGF or 30-day estimated glomerular filtration rate, has been reported^27^.

This study aimed to describe the effect of increased IAP on outcomes after transplantation, including its relationship with DGF, postoperative complications, a composite outcome of in-hospital graft failure or death and graft function recovery throughout the first 6 months post-transplantation.

## Materials and methods

### Study cohort and ethics

**Figure 1:**
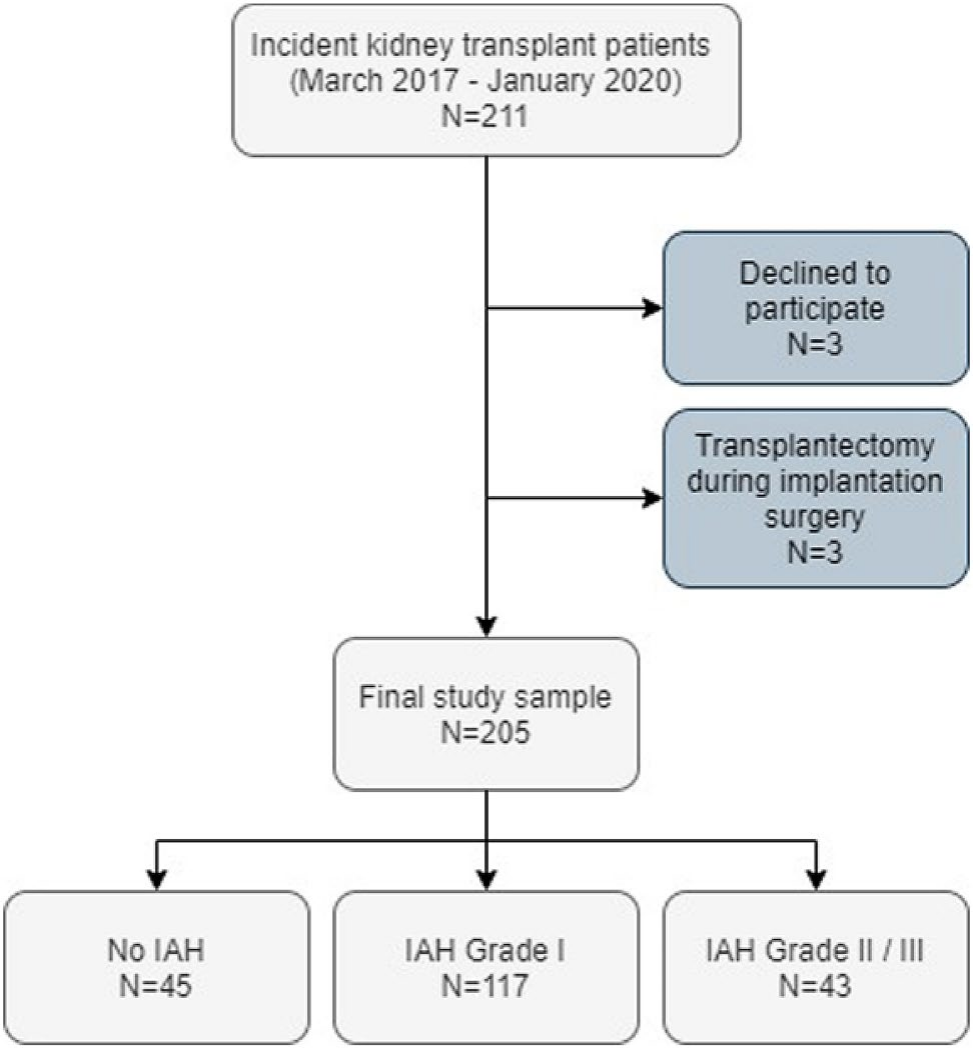
Study flow chart. *IAH* intra-abdominal hypertension.

This single-center prospective cohort study was conducted in the Nephrology Department of a tertiary referral university hospital (Hospital Clínico Universitario, Valladolid, Spain) from March 2017 to January 2020. During the study period, all consecutive patients ≥ 18 years-old who received a deceased-donor kidney transplant were asked to participate and give their written informed consent. The transplant unit where the study took place in does not perform living donor or multi-organ transplantation. Patients were excluded if they were unwilling to participate or if transplantectomy had to be performed during implantation surgery. Two hundred eleven patients received a kidney transplant in our unit during the study period. Three patients declined to participate. Transplantectomy had to be performed during implantation surgery in three cases due to incoercible hemorrhage. The final cohort included 205 kidney transplant recipients (Fig. 1). All patients were followed for 6 months after transplantation or until death or graft failure.

All data were collected prospectively. The study was designed in accordance with the ethical principles of the current Declaration of Helsinki. The protocol was approved by the Local Ethics Committee and the Institutional Review Board (CEIm Área de Salud Valladolid Este, PI18-1021).

### Surgical procedure and immunosuppressive medication

The anesthesia technique was the same in all cases. Surgery was performed according to local protocol. Allografts were perfused with cold University of Wisconsin solution and placed in cold storage. A modified Gibson or para-rectal incision with an extra-peritoneal approach of the iliac fossa was performed, with anastomosis of the renal vein to the external or common iliac vein, or inferior vena cava. The renal artery was anastomosed to the external iliac artery or common iliac artery. All vascular anastomoses were performed in an end‐to‐side fashion. No significant intra-abdominal adherences were found in any of the patients. All kidney allografts were placed in the retroperitoneal space. Anastomosis between donor ureter and recipient bladder was performed as described by Lich–Gregoir^28^. Ureterovesical double J stenting was used as standard procedure. All but two patients were weaned from ventilator support in the operation room and transferred to the nephrology unit. Hemodynamic monitoring during the first 72 h included measurement of mean arterial pressure, central venous pressure, blood loss volume, and urine output. Cold ischemia time was dichotomized using 16 h as a cut-off point, as described by Debout et al.^29^. Abdominal binders were not employed after transplantation.

Standard immunosuppressive medication typically consisted of basiliximab induction, corticosteroids in a tapering regimen and tacrolimus alone or in combination with mycophenolic acid or everolimus. Intra- and postoperative fluid administration (0.9% saline and dextrose 5% in water) was managed according to central venous pressure and hourly urine output. No cases of prolonged postoperative ileus were observed. No patient was treated with abdominal binders.

### Intra-abdominal pressure monitoring

We monitored IAP following WSACS guidelines^6,30^ using the UnoMeter Abdo-Pressure kit (Convatec Inc., Greensboro, USA), a closed sterile circuit that connects between the patient’s Foley catheter and the urine collection device. IAP measurement using the urinary bladder technique is currently considered the gold standard method for IAP monitoring^31^. The Foley manometer was placed between the catheter and the urine collection device and was primed with 20 mL of saline. With the patient in supine position, the “0 mmHg” mark of the manometer tube was placed at the symphysis pubis, the filter was elevated vertically above the patient and the clamp was opened. The end-expiration value for the bladder pressure was read after stabilization of the meniscus. After the measurement the clamp was closed, and the Foley manometer was placed in its drainage position. All measurements were performed after ensuring the absence of abdominal muscle contractions. The Foley catheter was kept in place for at least the first 72 h after transplant surgery in all cases. IAP values were registered immediately after transplantation and every 8 h during the first 72 h after surgery or until reoperation due to graft vascular thrombosis or other surgical complications. Overall, of the expected 1845 IAP measurements that should have been performed, 1788 (96.9%) were carried out.

Daily mean IAP, mean arterial pressure (average value obtained from three measurements), 24 h fluid balance and urine output values were calculated during the first 72 h after implantation surgery, which were defined as day-0, day-1 and day-2. 72 h average IAP, mean arterial pressure and accumulated fluid balance were calculated as the average of all available values measured during the first 72 h after surgery. IAH was graded according to WSACS recommendations (Grade I: IAP 12–15 mmHg; Grade II: IAP 16–20 mmHg; Grade III: IAP 21–25 mmHg; Grade IV: IAP > 25 mmHg)^6^. IAH was defined as the highest grade registered using daily mean values during the first 72 h after surgery for each subject. 72 h average IAP was chosen as a potential predictor of post-transplant outcomes rather than the first 24 h average IAP to consider a larger number of IAP measurements. Thus, we aimed to avoid potentially higher mean values that could be associated with surgical wound-associated pain, which is more common during the first hours after transplantation.

Measurements were performed by nurses from the transplant unit and not by the research team to ensure a pragmatic approach. All nurses received proper training in this procedure to reduce measurement bias. The frequency of measurements was not changed based on IAH diagnosis or severity.

### Renal resistive index measurement

RRI was measured using Doppler ultrasound 24 h after transplant surgery, employing a Canon Aplio a550 ultrasound system (Canon Medical Systems Europe BV, Zoetermeer, The Netherlands)^32^. All examinations were performed by trained radiologists. Peak systolic and end-diastolic velocities were measured in two or three representatives renal interlobar arteries. RRI was calculated using the following equation and the average values of all performed measurements:

$$ RRI = (peak\;systolic\;velocity - end\;diastolic\;velocity)/peak\;systolic\;velocity $$

### Data collection

Detailed demographic and clinical data such as age, chronic kidney disease etiology, time on dialysis or additional comorbidities, and donor information were gathered from the medical records. Hemodynamic parameters, laboratory and surgery-associated data were extracted from the patient’s charts and the electronic laboratory database.

### Outcomes

The primary outcome was to describe the impact of increased IAP on transplant results, such as DGF, postoperative complications, graft function recovery and a composite outcome of in-hospital graft failure or death. Secondary outcomes included:

1. Describing the prevalence of IAH immediately after surgery.
2. Characterize the association between mean IAP, RRI, mean arterial pressure, fluid balance and urine output during the first 24 h after surgery.

### Definitions

DGF was defined as the use of dialysis within seven days of transplantation. Graft function recovery was defined as reaching a SCr < 2.0 mg/dL off-dialysis^33^. Postoperative complications included need for re-intervention due to intra-abdominal hemorrhage, lymphocoeles or wound dehiscence (defined as Grade III-b or more severe according to the Clavien Dindo classification)^34^, graft artery or vein thrombosis or need for blood transfusion. The Kidney Donor Profile Index (KDPI) was calculated using the allocation calculator available at the US Organ Procurement and Transplantation Network website (https://optn.transplant.hrsa.gov/data/allocation-calculators/kdpi-calculator/).

### Statistical analysis

According to data distribution, demographics were summarized using mean and standard deviations (SD) or median and range for continuous variables and counts with percentages for binary variables. Continuous data were assessed using T-test, Mann–Whitney U test or Kruskal–Wallis test. Binary data were analyzed using the Chi-square test. Analysis of correlations was performed using Spearman Rho tests. A two-sided *P* value ≤ 0.05 was considered statistically significant. IAP measurements showed no data outliers upon screening with the Grubbs’ test (extreme studentized deviate method, http://graphpad.com/quickcalcs/grubbs1/).

Binary logistic regression analysis was used to find which variables were associated with IAH grade II/III, DGF and postoperative complications using odds ratios (OR) with 95% confidence intervals (CI). Cumulative survival curves of the 6-month follow-up were estimated with the Kaplan–Meier method, and the effect of IAH grade of graft function recovery was compared between groups using the log-Rank test. The Cox proportional hazards model with hazards ratios (HRs) with 95% confidence interval (CI) was used to evaluate potential risk factors of absence of graft function recovery. The proportional hazards assumption was tested using Schoenfeld residuals. Variables included in the adjusted regression models were selected due to their potential clinical relevance, considering potential multicollinearity issues, and a rate of at least 8 events per predictor variable. *P* values were adjusted for multiple comparisons using the Benjamini & Hochberg procedure.

Statistical analysis was carried out using the Statistical Package for Social Sciences software, version 20.0 (SPSS, IBM, Armonk, NY, USA), Stata version 14.1 (StataCorp LP, College Station, TX, USA) and GraphPad Prism, version 7.04 for Windows (GraphPad Software, La Jolla California USA).

## Results

### Baseline characteristics and IAH prevalence

The median age in the study sample was 61 years. 64.9% of patients were male. Subjects in the study cohort had spent a median of 28 months on dialysis before transplantation and presented an average body mass index (BMI) of 25.9 kg/m^2^. Median donor KDPI was 75%. Most patients (59.5%) had received hemodialysis as renal replacement therapy. In the whole cohort, the mean IAP value was 12.2 mmHg throughout the first 72 h after transplantation. Immediately after implantation, 94 subjects (45.9%) had IAP < 12 mmHg, 73 (35.6%) between 12 and 15 mmHg, 36 (17.6%) between 16 and 20 mmHg and 2 (1%) between 21 and 25 mmHg. During the first 24 h after surgery, 103 recipients (50.2%) had some form of IAH. According to maximal IAH grade achieved during the first 72 h after transplantation, 117 patients (57.1%) were classified as grade I IAH, 38 patients (18.5%) grade II IAH and five patients (2.4%) grade III IAH (Fig. 1). No patient suffered grade IV IAH.

### Predictors of postoperative IAH grade II/III

Subjects who suffered IAH during the early post-transplant period were more commonly males, had higher BMI, had spent a longer time on dialysis before transplantation and received more frequently hemodialysis as renal replacement therapy. Patients without IAH received organs from donors with lower KDPI (Table 1).

**Table 1:** Demographic, clinical characteristics and outcomes of transplant recipients according to the degree of IAH.

	All patients	No IAH	Grade I IAH	Grade II/III IAH
N	205	45	117	43
Age, years^1^	61 (51–69)	61 (48–69)	62 (52–70)	61 (53–68)
Male sex, n (%)	133 (64.9)	20 (44.4)	77 (65.8)	36 (83.7)
Caucasians, n (%)	202 (98.5)	45 (100)	116 (99.1)	41 (95.3)
Months on dialysis, months^1^	18 (10–33)	13 (7–37)	17 (9–31)	29 (13–43)
BMI, kg/m^2 2^	25.9 ± 3.9	23.6 ± 3.4	26 ± 3.7	27.9 ± 3.6
Polycystic kidney disease, n (%)	40 (19.5)	7 (15.6)	27 (23.1)	6 (14)
Peritoneal dialysis as RRT, n (%)	83 (40.5)	25 (55.6)	45 (38.5)	13 (30.2)
First renal transplant, n (%)	173 (84.4)	35 (77.8)	104 (88.9)	34 (79.1)
Hypertension, n (%)	181 (88.3)	36 (80)	107 (91.5)	38 (88.4)
Diabetes, n (%)	34 (16.6)	3 (6.7)	21 (17.9)	10 (23.3)
Age (donor), years^1^	60 (50–72)	56 (46–64)	62 (51–72)	60 (50–72)
Male sex (donor), n (%)	134 (65.4)	29 (64.4)	75 (64.1)	30 (69.8)
Donation after cardiac death, n (%)	16 (7.8)	4 (8.9)	8 (6.8)	4 (9.3)
Donor KDPI, percentage points^1^	75 (53–96)	63 (43–88)	78 (56–96)	78 (52–96)
Surgery duration, min^1^	185 (170–214)	173 (160–209)	190 (170–215)	193 (175–220)
Cold ischemia time, hours^1^	16 (13–19)	17 (14–19)	17 (13–19)	16 (13–18)
HLA mismatches, number^1^	4 (4–5)	5 (4–6)	4 (4–5)	4 (3–5)
Day 0 mean IAP, mmHg^1^	12 (9.7–14)	8.3 (7–10.2)	12 (10.5–13)	16.3 (15–18)
Day 1 mean IAP, mmHg^1^	12.3 (9.7–14)	9 (8–10)	12.7 (11–13.4)	15.8 (14–17)
Day 2 mean IAP, mmHg^1^	12.5 (10–14)	9.5 (8.4–10.7)	12.7 (11–13.7)	15.8 (13.7–17.3)
72 h mean IAP, mmHg^1^	12.1 (10.4–13.9)	9.1 (8.1–9.9)	12.1 (11.1–13)	15.6 (14.6–16.8)
72 h mean MAP, mmHg^1^	93.9 (90.8–97.6)	93.8 (90.6–96.7)	94.2 (91.1–98.1)	92.7 (90–97)
72 h accumulated fluid balance, L^1^	3.9 (2.6–5.1)	3.9 (2.6–4.9)	3.9 (2.4–5.1)	3.9 (2–5.6)
**Outcomes**				
Renal resistive index^1^	0.76 (0.7–0.83)	0.77 (0.7–0.82)	0.75 (0.7–0.8)	0.8 (0.73–0.88)
Graft failure or death at discharge, n (%)	13 (6.3)	1 (2.2)	6 (5.1)	6 (14)
Delayed graft function, n (%)	54 (26.3)	5 (11.4)	26 (22.6)	23 (54.8)
Postoperative complications, n (%)	86 (42)	20 (44.4)	41 (35)	25 (58.1)
SCr at discharge, mg/dL^1^	2 (1.51–3.19)	1.68 (1.33–2.22)	2.09 (1.51–3.03)	2.94 (1.8–4.62)
SCr at 3 months, mg/dL^1^	1.66 (1.31–2.14)	1.47 (1.16–1.78)	1.69 (1.33–2.2)	1.77 (1.42–2.65)
SCr at 6 months, mg/dL^1^	1.59 (1.29–2.01)	1.5 (1.17–1.69)	1.63 (1.29–2.1)	1.69 (1.44–2.05)
Days until SCr < 2.0 mg/dL^1^	17 (4–41)	8 (3–19)	17 (4–49)	25 (19–180)
Biopsy-proven Acute Rejection, n (%)	6 (2.9)	2 (4.5)	4 (3.5)	0 (0)
Length of stay, days^1^	14 (12–18)	13 (11.5–17.5)	14 (12–17)	16 (12–22)

^1^Reported as median (interquartile range).

^2^Reported as mean ± standard deviation.

*BMI* body-mass index, *HLA* human leukocyte antigen, *IAH* intra-abdominal hypertension, *KDPI* Kidney Donor Profile Index, *IAP* intra-abdominal pressure, *MAP* mean arterial pressure, *RRT* renal replacement therapy, *SCr* serum creatinine.

Recipient male sex, higher BMI and longer time on dialysis were independently associated with IAH grade II/III in adjusted binary logistic regression analysis. Although subjects that had received peritoneal dialysis as renal replacement therapy of choice suffered IAH less frequently, dialysis modality failed to predict IAH grade II/III (Table 2).

**Table 2:** Predictors of IAH grade II/III in the study cohort.

	Unadjusted analysis				Adjusted analysis			
	OR	95% CI		*P* value	OR	95% CI		*P* value
		Lower	Upper			Lower	Upper	
Recipient male sex	3.446	1.446	8.213	0.005	3.529	1.381	9.015	0.008
Months on dialysis, per month	1.009	1	1.019	0.051	1.012	1.003	1.022	0.014
BMI, per kg/m^2^	1.199	1.088	1.32	< 0.001	1.185	1.069	1.314	0.001
72 h accumulated fluid balance, per L	1	1	1	0.759	1	1	1	0.869
Peritoneal dialysis as RRT	1.756	0.854	3.612	0.126	1.622	0.71	3.704	0.251

*BMI* body mass index, *CI* confidence interval, *RRT* renal replacement therapy.

### Relationship between IAP, RRI, mean arterial pressure and fluid balance during the first 24 h after surgery

**Figure 2:**
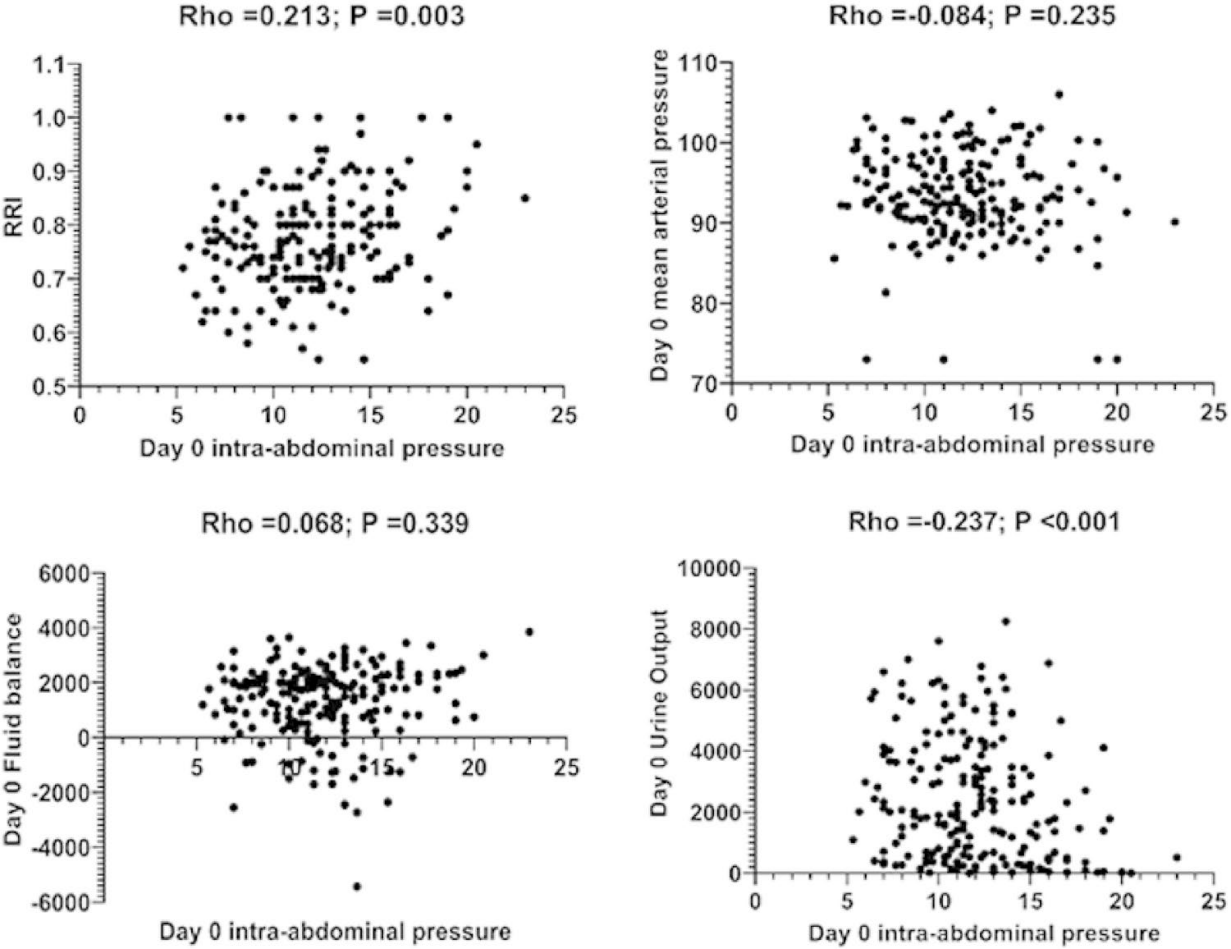
Association between Day-0 intra-abdominal pressure (IAP), renal resistive index (RRI), Day-0 mean arterial pressure, Day-0 fluid balance and Day-0 urine output. RRI (measured by Doppler ultrasound) and Day-0 urine output showed significant associations with Day-0 IAP (measured by the bladder method). No association of day-0 IAP with Day-0 mean arterial pressure and Day-0 fluid balance was evident.

Day-0 IAP was correlated with RRI and day-0 urine output but not with day-0 mean arterial pressure or fluid balance (Fig. 2). Recipient sex appears to modulate these correlations, with male subjects showing a stronger relationship between IAP, RRI and urine output than female patients [Day 0 IAP/RRI; Overall: Rho = 0.213; *P* = 0.003; Males: Rho = 0.222; *P* = 0.011; Females: Rho = 0.162; *P* = 0.184] [Day 0 IAP/Day 0 mean arterial pressure; Overall: Rho =  − 0.084; *P* = 0.235; Males: Rho =  − 0.016; *P* = 0.852; Females: Rho =  − 0.18; *P* = 0.137] [Day 0 IAP/Day 0 fluid balance; Overall: Rho = 0.068; *P* = 0.339; Males: Rho =  − 0.075; *P* = 0.511; Females: Rho =  − 0.086; *P* = 0.472] [Day 0 IAP/Day 0 urine output; Overall: Rho = 0.237; *P* < 0.001; Males: Rho =  − 0.343; *P* < 0.001; Females: Rho = 0.074; *P* = 0.538].

**Figure 3:**
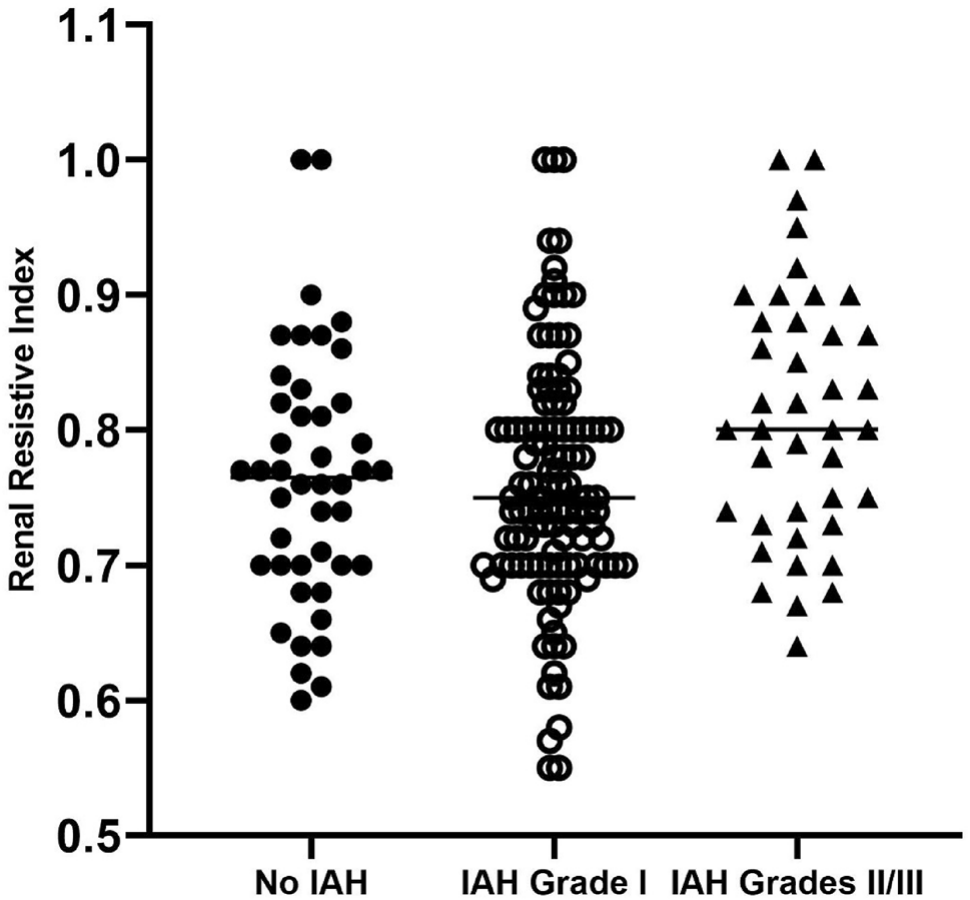
Stratification of renal resistive index (RRI) according to intra-abdominal hypertension (IAH) grade in renal transplant recipients. Subjects with IAH grades II/III presented significantly higher RRI (Kruskal–Wallis test). Overall: H = 8.01; *P* = 0.018. Male recipients (red): H = 3.18; *P* = 0.204. Female recipients (blue): H = 5.68; *P* = 0.058.

Patients who suffered IAH grades II and III had substantially higher RRI (Fig. 3).

### Intra-abdominal pressure and postoperative outcomes

Recipients with IAH experienced higher rates of DGF and postoperative complications and needed longer hospital length of stay (Table 1). In multivariable logistic regression analysis, 72 h mean IAP was the only variable independently associated with increased odds of postoperative complications (Table 3).

**Table 3:** Risk factors of postoperative complications in the study cohort.

	Unadjusted analysis				Adjusted analysis			
	OR	95% CI		*P* value	OR	95% CI		*P* value
		Lower	Upper			Lower	Upper	
Recipient age (per year)	1.017	0.994	1.04	0.148	1.024	0.996	1.053	0.09
Recipient male sex	1.018	0.569	1.821	0.952	0.677	0.347	1.321	0.253
Months on dialysis (per month)	1.005	0.996	1.013	0.287	1.004	0.995	1.014	0.351
Recipient BMI (per kg/m^2^)	1.033	0.962	1.11	0.372	0.993	0.914	1.078	0.86
Recipient diabetes	1.478	0.706	3.093	0.3	1.424	0.62	3.269	0.405
72 h mean MAP (per mmHg)	0.968	0.921	1.017	0.191	0.948	0.894	1.005	0.074
72 h mean IAP (per mmHg)	1.155	1.041	1.282	0.007	1.172	1.03	1.334	0.016

*BMI* body mass index, *IAP* intra-abdominal pressure (measured by the bladder method), *MAP* mean arterial pressure.

Out of five patients who suffered grade III IAH, three had to be reoperated due to renal vein thrombosis (two patients) or intraabdominal hemorrhage (one patient) and consequently lost graft function. Both subjects with renal vein thrombosis died due to postoperative complications. The two remaining grade III IAH patients died after hospital discharge due to cardiovascular complications and cytomegalovirus pneumonia.

In addition, duration of dialysis before transplantation and 72 h mean IAP were associated with increased odds of delayed graft function (Table 4).

**Table 4:** Risk factors of delayed graft function in the study cohort.

	Unadjusted analysis				Adjusted analysis			
	OR	95% CI		*P* value	OR	95% CI		*P* value
		Lower	Upper			Lower	Upper	
Recipient male sex	1.81	0.905	3.619	0.094	1.133	0.503	2.552	0.764
Months on dialysis (per month)	1.015	1.004	1.026	0.006	1.014	1.004	1.025	0.009
Cold ischemia time > 16 h	1.276	0.683	2.384	0.445	1.272	0.629	2.57	0.503
Donor KDPI > 75%	1.644	0.874	3.094	0.123	1.552	0.759	3.176	0.229
HLA mismatch ≥ 4	0.878	0.42	1.833	0.728	1.194	0.519	2.749	0.676
72 h mean IAP (per mmHg)	1.339	1.178	1.522	< 0.001	1.305	1.132	1.505	< 0.001

*HLA* human leukocyte antigen, *IAP* intra-abdominal pressure (measured by the bladder method), *KDPI* Kidney Donor Profile Index, *SCr* serum creatinine.

**Figure 4:**
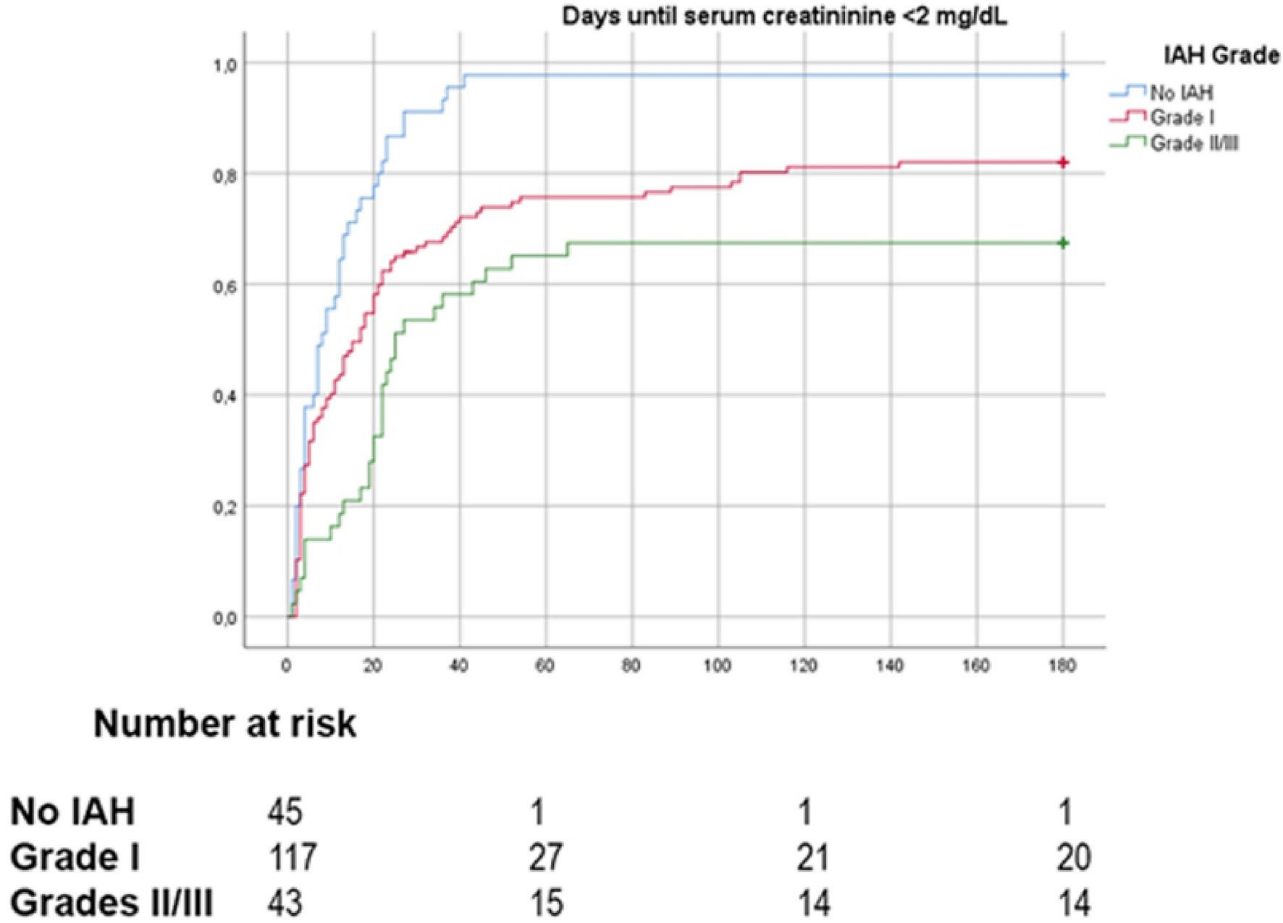
Kaplan–Meier plots of graft function recovery in patients stratified by intra-abdominal hypertension (IAH) grade.

IAH severity was associated with graft function recovery (log-Rank; Overall: 26.559, *P* value < 0.001; No IAH vs. IAH Grade I: 12.981, *P* value < 0.001; IAH Grade I vs. IAH Grade II/III: 5.530, *P* value = 0.019; No IAH vs. IAH Grade II/III: 27.931, *P* value < 0.001) (Fig. 4). This association was more evident among male subjects (log-Rank; Overall: 15.88, *P* value < 0.001) than among female patients. (log-Rank; Overall: 4.4, *P* value = 0.111).

72 h mean IAP was tested as a predictor for graft function recovery in Cox regression analysis. Higher 72 h mean IAP was independently associated with absence of graft function recovery (HR for recovery: 0.935, 95% CI: 0.875–0.998, *P* value = 0.044). DGF was also an independently associated with absence of graft function recovery (HR for recovery: 0.291, 95% CI: 0.191–0.445, *P* value < 0.001) (Table 5).

**Table 5:** Risk factors of graft function recovery in the study cohort.

	Unadjusted analysis				Adjusted analysis			
	HR	95% CI		*P* value	HR	95% CI		*P* value
		Lower	Upper			Lower	Upper	
Recipient male sex	0.554	0.404	0.758	< 0.001	0.685	0.483	0.97	0.033
Donor KDPI > 75%	0.742	0.548	1.006	0.055	0.915	0.666	1.256	0.581
Delayed graft function	0.253	0.168	0.381	< 0.001	0.291	0.191	0.445	< 0.001
Cold ischemia time > 16 h	0.873	0.645	1.182	0.38	0.965	0.705	1.391	0.823
72 h mean IAP (per mmHg)	0.87	0.821	0.921	< 0.001	0.935	0.875	0.998	0.044

*CI* confidence interval, *IAP* intra-abdominal pressure, *KDPI* Kidney Donor Profile Index.

Finally, patients with IAH grade II/III suffered more frequently a composite outcome of in-hospital death or graft loss (Table 1), although this relationship did not reach statistical significance in our sample. No multivariable analysis was performed due to the low number of events registered.

## Discussion

In the present study, we found that increased IAP after transplant surgery is a common event, affecting 78% of our recipients during the first 72 h after surgery. Furthermore, it is associated with DGF, postoperative complications and absence of recovery of graft function.

Half of the recipients (50.2%) developed IAH during the first 24 h after surgery, a proportion that increased to 78% when considering the first 72 h after transplantation. Although reported incidence and prevalence rates of IAH are highly variable, depending on the clinical setting, the method and frequency of measurements or the use of mean or maximum IAP values to define IAH, this was not an unexpected finding. The only evidence available regarding IAH and kidney transplant outcomes comes from a small study by Dupont et al., who have recently described that IAH occurred in 74.5% of 55 kidney transplant recipients in the early postoperative period^27^, a similar rate than that observed in our sample.

Higher BMI, male sex and longer time on dialysis were independently associated with IAH grade II/III in our cohort. BMI constitutes one of the strongest risk factors for IAH and its effect on IAP has been described in the critically ill, cardiothoracic surgical patients or those subjected to coronary artery bypass^35–37^. We found that male patients suffered more frequently IAH. Reintam-Blaser et al. reported a borderline significant association between male sex and IAH among critically ill patients^35^. Additionally, it has been recently described that female rats develop better hemodynamic response after exposure to increased IAP and higher survival than their male counterparts, suggesting a possible role of sex hormones in the hemodynamic adaptation to IAH^38^. Patients that received peritoneal dialysis as renal replacement therapy suffered IAH less commonly when compared to hemodialysis subjects, a finding that has also been described in another transplant cohort^27^. However, dialysis modality failed to predict IAH grade II/III in adjusted regression analysis in our study. Increased intra-peritoneal pressure, a surrogate of IAP is a common finding among peritoneal dialysis patients and has been related to several complications such as hernia or dialysate leakage^39,40^. Nevertheless, repeated peritoneal dialysis leads to abdominal wall expansion, explaining the lower prevalence of postoperative IAH among these patients. Additionally, intra-peritoneal pressure is directly correlated with actual infused volume^40^ and recipients are left with a dry abdomen immediately before transplant surgery, thus removing the main driving determinant behind increased intra-peritoneal pressure in this subset of subjects.

Other factors, such as aggressive fluid resuscitation, mechanical ventilation, abdominal infection, or distension have been previously described as significant risk factors of IAH^14,27,41–43^. Only two patients required mechanical ventilation for 48 h or more after surgery and none suffered clinically significant abdominal distension or infection. Aggressive fluid administration was common in our sample, with average 72 h fluid gains over 3.5 L. Nevertheless, fluid balance was not associated with IAH in our study. As Mohmand et al.^41^ proposed, several conditions that are common among the critically ill, but not in kidney transplant recipients, such as sepsis, coagulopathy or polytransfusion could increase capillary permeability and fluid accumulation in the interstitium, facilitating the development of fluid balance-associated IAH. These factors may be common in the ICU setting but are considerably rarer after kidney transplantation.

IAH may occur in the transplant setting due to the joint action of transplant- and patient-related factors^27^. Reduction of abdominal wall compliance due to recent abdominal surgery, increased intra-luminal and intra-abdominal contents due to postoperative ileus, abdominal distension, and graft placement in the retroperitoneal space may facilitate the rise of IAP. In addition, patient-related risk factors, such as high BMI and certain pre-existing pathologies such as hypertension or diabetes that may act in conjunction with common complications in kidney transplant patients such as metabolic acidosis, facilitating the development of ischemia–reperfusion associated endothelial dysfunction and capillary leakage, which may further exacerbate the growth of IAP. Endothelial dysfunction is closely related to chronic kidney disease. Certain risk factors for endothelial dysfunction, such as chronic volume overload, anemia, abnormal calcium-phosphate metabolism, increased oxidative stress and inflammation^44^, are common among subjects with end-stage chronic kidney disease. Several of these factors have also been linked to the development of IAH^45^. Longer dialysis duration may act in this setting as a surrogate marker of the maintenance of these risk factors over time.

Besides, we found that 72 h mean IAP was independently associated with DGF in adjusted binary logistic regression analysis. 72 h mean IAP also predicted graft function recovery in adjusted Cox regression analysis. These results support the notion that higher IAP not only exerts a pernicious effect on outcomes immediately after surgery but can also affect graft function days after transplantation. IAH grades III/IV have been associated with increased risk of DGF and lower day-30 eGFR in a previous report^27^. However, contrary to what has been done in that study, we chose to use IAP as a continuous rather than categorical predictor variable because IAH and abdominal compartment syndrome only represent specific cut-off points on a spectrum of increasingly harmful pressure that can alter intra-abdominal tissue perfusion and reduce organ viability^19^. Moreover, these cut-off points were developed using data from critically ill subjects and were not specifically designed for the transplant setting. We also chose to extend follow-up in our cohort up to the first 6 months after transplantation to analyze if IAP might influence graft function beyond immediate recovery.

Although the pathological processes involved are not completely understood, several IAP-induced kidney injury mechanisms have been proposed, such as direct parenchymal and vascular compression or exacerbation of renal tubular necrosis^41^. Indeed, we observed that higher IAP during the first 24 h after transplantation was significantly associated with higher RRI and lower urine output during that timeframe. RRI measurement, an easy-to-reproduce Doppler ultrasound study, has been used to predict AKI in ICU and postoperative settings^46,47^, as well as DGF, acute rejection or long-term mortality in renal transplant recipients^48–50^. We hypothesize that, in the early post-transplant period, augmented IAP may compress the inferior vena cava and renal vasculature, reducing cardiac output and venous return while increasing the RRI, a relationship that has already been observed in humans^24^. As a consequence, the reduction in renal blood flow can extend in time the post-operative acute tubular necrosis, impairing the urine output, facilitating the development of DGF, and providing a pathological link between increasing IAP and absence of recovery of kidney graft function. Nevertheless, an organ-centric view of the RRI should be avoided; the index is, in fact, the product of multiple interactions between hemodynamic, renal and systemic vascular factors, many of which may not be correlated with IAP.

Of note, 72 h mean IAP was the only variable independently associated with postoperative complications in adjusted regression analysis. Low urine output can be common early after transplantation, especially in patients with little to no pretransplant residual renal function, while transient hypotension and mild or moderate abdominal pain are frequent findings in this setting. Close IAP monitoring can offer, in combination with the rest of hemodynamic parameters and clinical findings, an early warning sign in case of postoperative complications that may allow faster diagnostic and therapeutic approaches immediately posttransplant. Short-term results after transplantation have vastly improved in the last decades thanks to advancing of surgical techniques and the optimization of immunosuppression therapy. However, these improvements are marred by the inclusion of older and higher-risk recipients in transplant programs. IAP measurements might help define recipients at greater risk of postoperative complications and/or absence of graft function recovery, to adopt preventive measures aimed to minimize this risk, such as tighter monitoring of immunosuppressive drug levels to avoid potential nephrotoxicity or repeated imaging tests to control intra-abdominal bleeding.

The present study entails limitations. Its single-centered nature potentially limits external validity. The study sample was mostly composed of Caucasians, limiting the extrapolation of results to other ethnic groups. Furthermore, WSACS guidelines advocates for IAP measurement every 4–6 h in critically ill patients. However, the department where the study was conducted was not an ICU and we chose to measure IAP every 8 h to facilitate patient care. Finally, the severity of IAH observed in this study was, in most cases, mild or moderate. Very few patients developed severe IAH, with only five subjects suffering maximal IAH grade III and none developing IAH grade IV. As such, our study is not sufficiently powered to adequately describe the effects of IAH grades III/IV on early transplant outcomes.

However, we also recognize several strengths. This is one of the first prospective studies designed to analyze the consequences of increased IAP early after transplantation. We applied WSACS consensus guidelines in kidney transplant patients using a standardized and widely accepted method to measure IAP. The extended follow-up enabled us to describe the influence of IAP on DGF and graft function recovery and early posttransplant complications. Measurements were performed by trained nurses instead of research staff to increase their real-world applicability and to avoid relying on personnel with high commitment or experience in this subject. Most planned IAP measurements (> 96%) were completed, reflecting high compliance rates to the protocol. IAP measurement is an easy to implement and standardized process that requires only bladder catheterization. Systematic measurement of IAP during the first hours after surgery may allow a faster diagnosis of severe postoperative complications that could affect graft and patient survival, allowing early treatment initiation and improving results after transplantation.

In sum, the present study describes increased IAP as a promising predictor of postoperative complications and absence of renal function recovery in the early kidney post-transplant period. The present report complies with WSACS guidelines using a pragmatic approach in a prospective cohort of incident kidney transplant recipients. Future studies should analyze the impact of IAP-guided interventions on the early postoperative care of kidney transplant recipients and their impact on patient and graft outcomes.

## Abbreviations

BMI: Body mass index
CI: Confidence interval
DGF: Delayed graft function
HLA: Human leukocyte antigen
HR: Hazard ratio
IAH: Intra-abdominal hypertension
IAP: Intra-abdominal pressure
ICU: Intensive care unit
MAP: Mean arterial pressure
OR: Odds ratio
RRI: Renal resistive index
RRT: Renal replacement therapy
SCr: Serum creatinine
WSACS: World Society of Abdominal Compartment Syndrome
